# Resistant starch supplementation increases crypt cell proliferative state in the rectal mucosa of older healthy participants

**DOI:** 10.1017/S0007114520001312

**Published:** 2020-04-13

**Authors:** Fiona C. Malcomson, Naomi D. Willis, Iain McCallum, Long Xie, Arthur C. Ouwehand, Julian D. Stowell, Seamus Kelly, D. Michael Bradburn, Nigel J. Belshaw, Ian T. Johnson, John C. Mathers

**Affiliations:** 1 Human Nutrition Research Centre, Population Health Sciences Institute, Newcastle University, Newcastle upon Tyne NE2 4HH, UK; 2 Northumbria Healthcare NHS Foundation Trust, North Shields NE29 8NH, UK; 3 DuPont Nutrition & Biosciences, 02460 Kantvik, Finland; 4 DuPont Nutrition & Biosciences, Reigate RH2 9PQ, UK; 5 Northumbria Healthcare NHS Foundation Trust, Ashington NE63 9JJ, UK; 6 University of East Anglia, Norwich Research Park, Norwich NR4 7TJ, UK; 7 Quadram Institute, Norwich Research Park, Norwich NR4 7UQ, UK

**Keywords:** Dietary fibre, Resistant starch, Polydextrose, Crypt cell proliferation, Apoptosis, Colorectal cancer risk

## Abstract

There is strong evidence that foods containing dietary fibre protect against colorectal cancer, resulting at least in part from its anti-proliferative properties. This study aimed to investigate the effects of supplementation with two non-digestible carbohydrates, resistant starch (RS) and polydextrose (PD), on crypt cell proliferative state (CCPS) in the macroscopically normal rectal mucosa of healthy individuals. We also investigated relationships between expression of regulators of apoptosis and of the cell cycle on markers of CCPS. Seventy-five healthy participants were supplemented with RS and/or PD or placebo for 50 d in a 2 × 2 factorial design in a randomised, double-blind, placebo-controlled trial (the Dietary Intervention, Stem cells and Colorectal Cancer (DISC) Study). CCPS was assessed, and the expression of regulators of the cell cycle and of apoptosis was measured by quantitative PCR in rectal mucosal biopsies. SCFA concentrations were quantified in faecal samples collected pre- and post-intervention. Supplementation with RS increased the total number of mitotic cells within the crypt by 60 % (*P* = 0·001) compared with placebo. This effect was limited to older participants (aged ≥50 years). No other differences were observed for the treatments with PD or RS as compared with their respective controls. PD did not influence any of the measured variables. RS, however, increased cell proliferation in the crypts of the macroscopically-normal rectum of older adults. Our findings suggest that the effects of RS on CCPS are not only dose, type of RS and health status-specific but are also influenced by age.

## Graphical abstract

**Figure:**
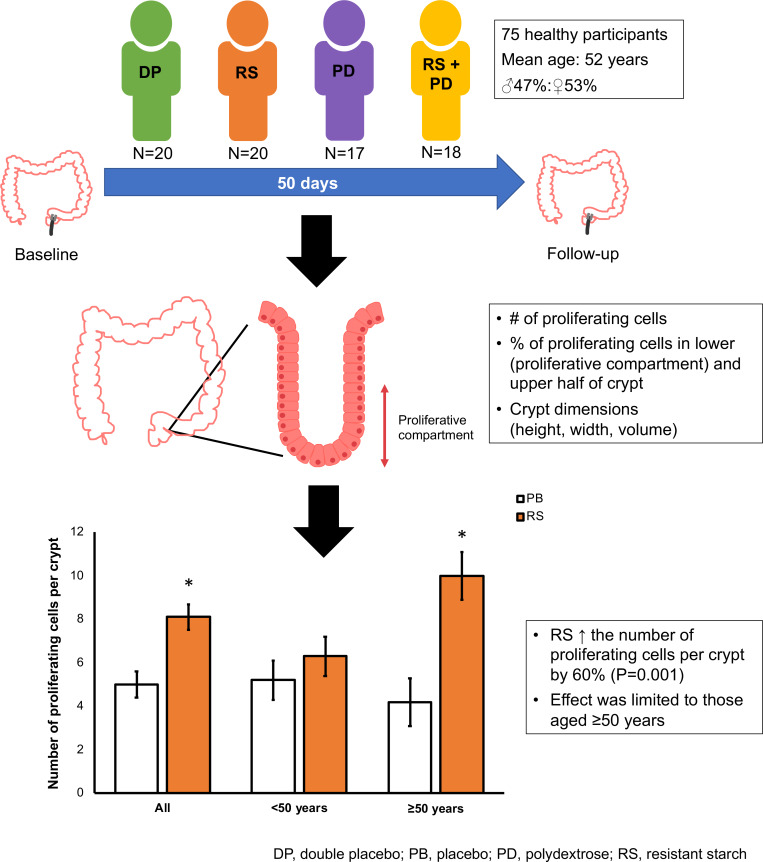

There is strong evidence for a protective effect of foods containing dietary fibre against colorectal cancer (CRC)^(1,2)^. This protection may result from the production of the anti-proliferative SCFA, butyrate, the primary energy source for colorectal epithelial cells, as an end product of dietary fibre fermentation in the large bowel. Dietary fibre is a collective term for a physiochemically-diverse group of non-digestible carbohydrates (NDC), including starches that resist small bowel digestion (resistant starch; RS) and synthetic NDC such as polydextrose (PD)^(3,4)^. Hi-Maize® 260 is a type 2 RS, containing 46 % RS, that is highly fermentable and produces relatively large amounts of butyrate when compared with other RS-rich forms of high-amylose maize starches^(5)^. PD is a glucose polymer, produced from sorbitol, dextrose and citric acid, of which up to 50 % is fermentable^(3,6)^.

In an attempt to investigate the possible relationship between RS intake and CRC risk, Cassidy *et al.* showed that there was a strong inverse relationship between total starch intake and CRC risk using data from twelve countries worldwide^(7)^. The authors suggested that RS may contribute at least 5 % of total starch intake and, on that basis, a corresponding inverse relationship between RS intake and CRC risk would be expected^(7)^. Although the RS content of foods can be assessed using a standardised enzyme-based method^(8)^, most food composition databases do not contain comprehensive RS values for foods and this limits the potential for relevant epidemiological studies of RS intake and CRC risk. However, in a number of case–control studies carried out in Italy, higher starch intakes were associated with greater CRC risk^(9–11)^. Also in Italy, a starch-rich dietary pattern was associated with greater CRC risk in both those with and without a family history of CRC^(12)^. In contrast, there was no significant relationship between starch intake and CRC risk in a case–control study in the Swiss Canton of Vaud^(13)^ or in a case–control study in China^(14)^. Few studies have investigated links between starch intake and risk of colorectal adenomas and, for example, there was no association between starch intake and adenoma risk in the Tennessee Colorectal Polyp Study^(15)^. The findings from these studies of total starch intake should be interpreted with caution because of lack of data on RS intake and the potential for confounding by other dietary (and lifestyle) factors.

The evidence for relationships between individual NDC, such as RS, and biomarkers of colorectal carcinogenesis, such as DNA damage, crypt cell proliferation and aberrant crypt foci formation is inconsistent^(16)^. While a number of studies suggest a chemoprotective effect of RS on these markers^(17–20)^, others have reported no effect^(21,22)^ and some studies in animals have reported stimulated epithelial proliferation and aberrant crypt foci formation^(23)^ and increased small intestinal tumours^(22)^ with RS feeding.

Loss of regulation of cell kinetics, resulting in an imbalance between mitosis and apoptosis in favour of a hyperproliferative state, is associated with increased risk of colorectal carcinogenesis^(24)^. Furthermore, the distribution of mitotic cells within the crypt is important. In healthy colorectal mucosa, the large majority of cell division occurs in the basal half of the crypt and the offspring cells differentiate as they migrate upwards towards the gut lumen. A higher proportion of proliferating cells in the upper half of the crypt is one of the earliest detectable alterations in the apparently normal mucosa prior to the development of malignancy^(25)^. Expansion of this ‘proliferative compartment’ is considered a pre-neoplastic biomarker and is observed even in the apparently-normal mucosa of patients with adenomas or CRC^(26,27)^.

Dietary interventions investigating the effects of RS on crypt cell proliferative state (CCPS) have yielded diverse results, and few studies have been carried out in healthy individuals^(16)^. In an early study with fourteen healthy participants, feeding 28 g RS type 2 (45 g native amylomaize; Hylon VII) for 2 weeks reduced total mitoses by approximately 20 %^(17)^. In contrast, no significant differences in mitosis or in the distribution of proliferating cells within the crypt were reported when healthy volunteers were fed a RS-enriched diet (supplemented with Hylon VII) compared with a low-RS diet for 4 weeks^(28)^ or 12·5 g RS/d for 4 weeks^(29)^. In one study, a 2-week food exchange was performed between African Americans and rural Africans^(30)^. At baseline, dietary fibre (mainly in the form of RS) intake was significantly greater in rural Africans, who also had significantly lower mucosal epithelial proliferation rates. In the African Americans given the African high-fibre diet for 2 weeks, cell proliferation reduced to levels similar to those observed in Africans on their habitual diet^(30)^. Studies in CRC patients or in those at greater risk of CRC, for example, those with sporadic adenomas, have reported no significant effect on total mitoses of supplementation with RS, for up to 12 years^(31)^. However, we observed that supplementation with a 1:1 blend of RS type 2 and type 3 for up to 4 weeks reduced the proportion of mitotic cells in the top half of the crypt of CRC patients^(32)^.

Very few studies have investigated the effects of PD on CCPS. In healthy humans supplemented with PD (4, 8 or 12 g PD/d) for 28 d, PD increased total mitoses in the caecal mucosa^(33)^. Following high-dose PD feeding (20 % of diet) of Sprague–Dawley rats, crypt length and total mitoses in the Caecum were increased^(34)^. However, exposure of Caco-2 human colonic adenocarcinoma cells *in vitro* to media containing the end products of PD fermentation reduced cell proliferation^(35)^.

In healthy colorectal epithelia, levels of apoptosis are relatively low but are increased in response to DNA damage^(36)^. Reduced apoptosis, or the evasion of apoptosis, is a hallmark of colorectal carcinogenesis and can result from abnormal expression of regulators such as *BAX* (BCL2 associated X; pro-apoptotic) and *BCL-2* (B-cell lymphoma 2; anti-apoptotic). In carcinogen-treated mice, RS increased apoptosis rates and reduced expression of Bcl-2 and p53, as well as reduced cell proliferation and differentiation, and this was associated with reduced incidence of colonic tumours^(37)^. Similarly, RS feeding led to greater expression of *Bax* and reduced *Bcl-2* expression in carcinogen-treated mice^(38)^. *In vitro* treatment of Caco-2 cells with media containing the end products of PD fermentation increased apoptosis, suggesting that PD may have chemoprotective effects in cancer cells by inducing apoptosis^(35)^. The effects of supplementation with RS or PD on apoptosis in the human colorectal mucosa are unknown.

This is the first study to investigate the effects of supplementing healthy human adults with RS and PD (separately and in combination) on crypt cell dynamics in the macroscopically normal rectal mucosa and to investigate relationships between expression of regulators of the cell cycle, and of apoptosis, with measures of crypt cell proliferation. Furthermore, it is the longest study (50 d) to investigate the effects of RS on cell proliferation in healthy individuals (previous studies have had an intervention duration of maximum 4 weeks^(28,29,39)^). On the basis that higher dietary fibre intakes are associated with lower CRC risk, we hypothesised that supplementing with RS and/or PD would reduce biomarkers of CRC risk. Such evidence would help to inform public health advice about links between dietary fibre intake and CRC risk.

## Methods

### Study design

Participants were recruited to the Dietary Intervention, Stem cells and Colorectal Cancer (DISC) Study, described previously^(40)^, a randomised, placebo-controlled dietary intervention that investigated the effects of RS and PD using a 2 × 2 factorial design. The present was conducted according to the guidelines laid down in the Declaration of Helsinki, and all procedures involving human subjects were approved by the Newcastle and North Tyneside Research Ethics Committee on 10 December 2009 (REC No. 09/H0907/77). Caldicott approval for the storage of data was given by the Northumbria NHS Foundation Trust (C1792). The DISC Study is registered with ClinicalTrials.gov (NCT01214681). Seventy-five healthy volunteers were recruited from gastroenterology out-patients departments at North Tyneside General Hospital, North Shields, UK and Wansbeck General Hospital, Ashington, UK between May 2010 and July 2011. Written informed consent was obtained from all subjects. The exclusion criteria were aged <16 or >85 years, a prisoner at the time of endoscopy, pregnant or planning to become pregnant, diabetes mellitus, familial adenomatous polyposis syndrome, Lynch Syndrome, known colorectal tumour or prior CRC, prior colorectal resection, active colonic inflammation at endoscopy, iatrogenic perforation at endoscopy, incomplete left-sided examination, colorectal carcinoma discovered at endoscopy, CRC on histology, chemotherapy in the last 6 months, administering non-steroidal anti-inflammatories, for example, aspirin, anti-coagulants, for example, warfarin or immunosuppressive medication, for example, methotrexate.

At least 1 week post-baseline endoscopy, participants were randomised to receive RS (23 g Hi-maize® 260, Ingredion™, Food Innovation), PD (12 g of Litesse® *Ultra*™ DuPont™ Danisco®), RS and PD or double placebo (12 g of Maltodextrin (RS placebo) and 23 g of Amioca starch (PD placebo)) for 50 d in a 2 × 2 factorial design. Randomisation was stratified by pre-intervention endoscopy procedure (see below).

Anthropometric measurements (weight, height, waist circumference and hip circumference) were collected and BMI was calculated. Habitual diet was assessed using a FFQ adapted from that used in the European Prospective Investigation into Cancer and Nutrition Study^(41,42)^. Dietary fibre intake was calculated using the Southgate method^(43)^. Biological samples including blood, urine, stool and rectal mucosal biopsies (10 cm from the ano-rectal verge) were collected at baseline (pre-intervention) and post-intervention. In addition, anthropometric measurements were made, and we collected information on physical activity, health history and smoking. Pre-intervention rectal mucosal biopsies were collected by colonoscopy or flexible sigmoidoscopy and post-intervention rectal mucosal.

Participants were asked to provide stool samples pre- and post-intervention. Pre-intervention samples were collected at least 7 d after their initial endoscopy appointment to allow for washout after the bowel preparation and for complete turnover of cells in the colorectal epithelium. Second samples were collected just prior to the participant’s repeat sigmoidoscopy after 50 d of dietary intervention. For each collection, participants were provided with a large sealable bucket pot, a disposable bedpan, two ice packs and a cool bag. Participants were instructed to freeze the ice packs at home for at least 24 h before sample collection. Following collection of stool samples, they were kept cold in the cool bags containing the ice packs and either picked up by a member of the research team (pre-intervention samples) or brought by the participant to the clinic (post-intervention samples). As soon as possible, and always on the same day as the stool samples were collected, samples were divided into five or six separate aliquots (in 20 ml universal tubes) and archived immediately at −80°C until analysis.

### Assessment of crypt cell proliferation state

CCPS was assessed in whole microdissected, Schiff reagent-stained crypts as described by Mills *et al.*^(44)^. Carnoy’s fixed rectal mucosal biopsies were hydrated in 50 % ethanol for 10 min at room temperature. This was repeated with 25 % ethanol. Biopsies were then hydrolysed in 1m HCl for 10 min at 60°C. Samples were stained by incubating with Schiff reagent (Surgipath™) for 1 h at room temperature. The Schiff reagent was removed and replaced with 1 ml of 45 % acetic acid. Whole crypts were microdissected using an Olympus SZ40 dissecting microscope and Leica CLS 150X light source by placing the stained biopsies on a microscope slide (Surgipath™, Leica Microsystems) with a drop of 45 % acetic acid (bases of the crypts facing upwards) and teasing apart rows of individual crypts using fine gauge hypodermic needles (25 G × 5/8′′; Terumo®). Any connective tissue or Peyer’s patches were removed, and the crypts were covered and sealed with a cover slip (Surgipath®, Leica). Under a light microscope at 40× magnification, ten intact crypts were selected at random. Each crypt was divided longitudinally into ten equal compartments and the number of mitotic cells in each compartment was counted, starting from the base of the crypt. Mitotic cells were those in prophase, metaphase, anaphase or telophase. The proportion of mitotic cells in the top half of the crypt (the top five compartments) was determined. In addition, crypt width and length were measured using a graticule.

### Quantification of expression of cell cycle regulators *(CCND1, c-MYC* and *c-JUN)* and regulators of apoptosis *(BAX* and *BCL-2)*

RNA was extracted from rectal mucosal biopsies using the RNeasy Mini Kit (Qiagen) and the miRNeasy Mini Kit (Qiagen), as described by the manufacturer. Tissue disruption was performed by shaking the tissue samples with five 3 mm glass beads (VWR) for 1 min in 150 μl of Buffer RLT (RNeasy Mini Kit) or 700 µl QIAzol Lysis Reagent (miRNeasy Mini Kit) using an amalgamator. Tissue homogenisation was performed using QiaShredders (Qiagen). RNA concentrations and purity were assessed using the NanoDrop 1000 spectrophotometer (Thermo Scientific) and NanoDrop 1000 Software version 3.7.1 and RNA integrity was examined by agarose gel electrophoresis. RNA was reverse transcribed to cDNA using the QuantiTect Reverse Transcription Kit (Qiagen) as described by the manufacturer and diluted 10× in nuclease-free water.

Quantification of cyclin D1 (*CCND1*), *c-MYC* (v-myc avian myelocytomatosis viral oncogene homolog) and *SFRP1* (secreted frizzled-related protein 1), together with two reference genes *18S* and *B2M*, was performed using primers designed and optimised by Dr Nigel Belshaw and Dr Wing Leung (Quadram Institute) (please find primer sequences in online Supplementary Table S1). Each reaction mix contained 5 µl ImmoMix™ (2×) (Bioline), 0·1 µl MgCl_2_ (50 mm) (Bioline), 1 µl bovine serum albumin (10 mg/ml) (Ambion), 0·2 µl ROX Reference Dye (50×) (Invitrogen), 0·06 µl SYBR Green (100×) (Invitrogen), 0·6 µl RNase-free water, 0·02 µl each of forward and reverse primers (100 µm) and 3 µl of cDNA. The programme was run for a 10 min activation step at 95°C followed by forty cycles of 30 s each, denaturation at 95°C, annealing at 60°C and extension at 72°C. Expression of *BAX* and *BCL-2* and of two reference genes, *18S* and *β2M*, was measured by quantitative PCR using the QuantiFast SYBR® Green PCR Kit (Qiagen) and QuantiTect® primer assays (*18S*: Hs_RRN18S_1_SG, *β2M:* Hs_B2M_1_SG, *BAX:* Hs_BAX_1_SG, *BCL-2*: Hs_BCL2_1_SG) as described by the manufacturer. Quantitative PCR analyses were performed using the Applied Biosystems StepOnePlus system.

### Quantification of faecal SCFA concentrations

Volunteers collected a faecal sample prior to the start of the intervention and at the end of the intervention.

Samples were refrigerated and transported to the laboratory within 16 h to be frozen and stored below –18°C. Gas chromatographic analysis of the SCFA (acetic acid, propionic acid and butyric acid) was performed using pivalic acid as an internal standard as described previously^(45)^. In short, 1 ml 20 mm pivalic acid and 5 ml water were added to 1 g of faecal sample. After thorough mixing, the sample was centrifuged at 5000 ***g*** for 5 min. Following centrifugation, 0·250 ml saturated oxalic acid solution was added to 0·5 ml of the supernatant fraction and the mixture was incubated at 48°C for 60 min, then centrifuged at 16 000 ***g*** for 5 min. The supernatant fraction was analysed by GC as described previously^(46)^.

### Statistical analyses

The study was not subject to a formal power calculation, and a target of seventy-five participants, allowing for a 10 % dropout rate, was set based on our previous study which detected significant effects of supplementation with RS for <4 weeks on CCPS in sixty-five CRC patients^(32)^.

This study was designed as a 2 × 2 factorial so to estimate the effect of RS, we compared the mean of the two groups that received RS (intervention groups C and D in Table 1; *n* 17 + 18 = 35) with that of the two groups that did not receive RS (intervention groups A and B in Table 1; *n* 20 + 20 = 40). The same principle applied when testing the effect of PD so that thirty-eight participants received PD and thirty-seven received the corresponding placebo. Statistical analyses were performed using Minitab® v17.1.0. The ANOVA general linear model (GLM) was used to test for effects of the intervention agents RS and PD, and for interactions between these two NDC, on the measured markers of CCPS and on the expression of the regulators of the cell cycle and apoptosis. Age, sex, BMI, endoscopy procedure, pre-intervention measurement and smoking status were included as covariates to adjust for baseline differences between intervention groups. Where data were not normally distributed despite transformation, the non-parametric Mann–Whitney test was applied. Differences between the younger and older participants (dichotomised at age 50 years) were investigated using independent *t* tests or Mann–Whitney. Potential relationships between gene expression and faecal SCFA concentrations and CCPS outcomes were investigated using Spearman’s rank correlation test.

**Table 1.** Characteristics of Dietary Intervention, Stem cells and Colorectal Cancer study participants (Mean values and ranges; numbers of participants; ratios; percentages)*

Intervention group...	A			B			C			D		
	Mean		Range	Mean		Range	Mean		Range	Mean		Range
Resistant starch	**–**			**–**			**+**			**+**		
Polydextrose	**–**			**+**			**–**			**+**		
*n*		20			20			17			18	
Age (years)	48		30–70	58		33–80	53		42–67	50		36–74
Sex (males:females)		10:10			11:9			13:4			6:12	
Ethnicity (*n*)												
Caucasian		19			19			17			18	
Black African		1			0			0			0	
Mixed race		0			1			0			0	
Endoscopy procedure (%)												
Colonoscopy		30			30			35			28	
Flexible sigmoidoscopy		70			70			65			72	
BMI (kg/m^2^)	29·8		23–37	29·7		23–49	30·9		23–43	29·9		23–42
Smoking status (*n*)												
Never		12			12			8			6	
Former		4			5			6			6	
Current		4			3			3			6	

* Data are presented as number of participants (*n*) with the exception of age and BMI presented as mean (range) and endoscopy procedure presented as proportion of participants (%).

## Results

### Participant characteristics

Seventy-five healthy participants completed the 50-d dietary intervention (Table 1). Just over half of participants were male. Further characteristics of the DISC Study participants are reported by Malcomson *et al.*^(40)^.

### Effects of resistant starch and polydextrose on crypt cell proliferative state and crypt dimensions

**Fig. 1.**
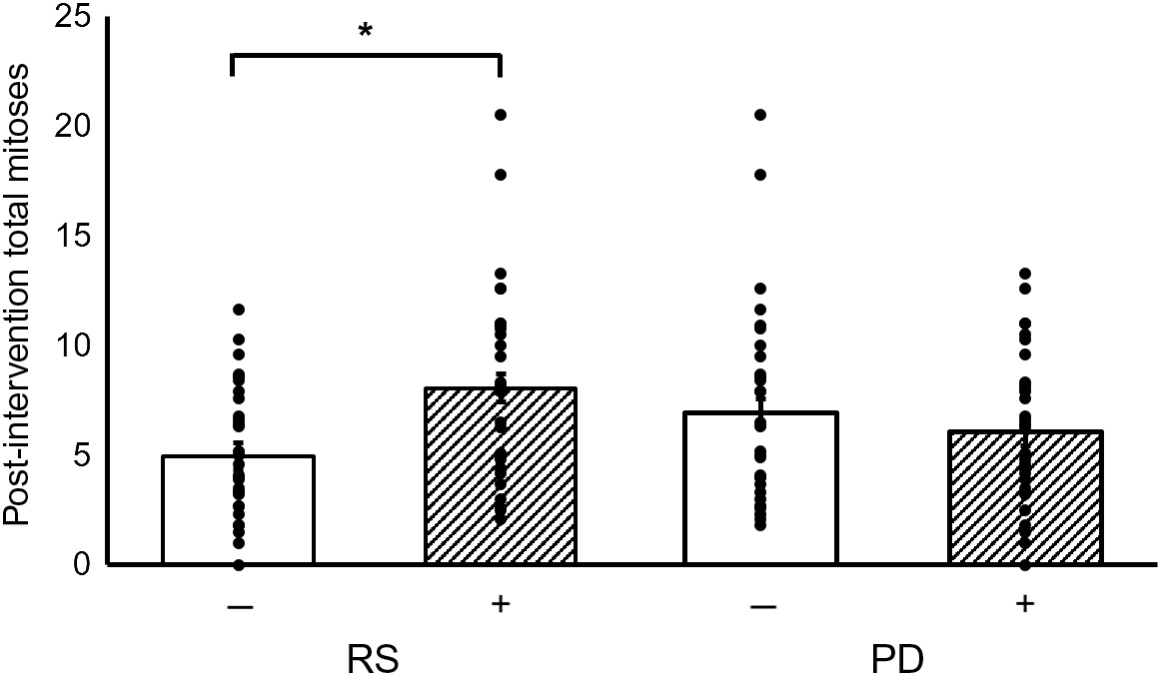
Effects of resistant starch (RS) and polydextrose (PD) on total mitoses post-intervention. Data are presented as individual data plots, and bars represent least squares means for data adjusted for pre-intervention total mitoses, age, sex, endoscopy procedure, BMI and smoking status (ANOVA general linear model (GLM)). Error bars represent standard errors of the mean. * Significant effect of the intervention (*P* < 0·05).

Supplementation with RS increased the total number of mitotic cells within the crypt by 60 % (*P* = 0·001) (Fig. 1). However, there were no statistically significant effects of PD on total mitoses or of RS or PD on the distribution of the mitotic cells or on crypt dimensions (length, width or volume) (Table 2). Furthermore, there were no significant interactions between RS and PD on the measured outcomes (Table 2).

**Table 2.** Effects of resistant starch (RS) and polydextrose (PD) on crypt cell proliferative state (CCPS) and crypt dimensions (Least squares means (LSM) with their standard errors; medians and interquartile ranges (IQR))

CCPS marker	Effect of RS								*P*	Effect of PD								*P*	Interaction between RS and PD: *P*
	**–**				**+**					**–**				**+**					
	LSM	Median	IQR	sem	LSM	Median	IQR	sem		LSM	Median	IQR	sem	LSM	Median	IQR	sem		
Total mitoses†	4·98			0·61	8·06			0·63	0·001*	6·96			0·63	6·08			0·63	0·336	0·684
Proportion (%) of mitotic cells in the top half of the crypt‡		7·8	10·2			6·9	7·8		0·733		7·3	9·8			6·6	7·8		0·732	0·613
Crypt length (μm)‡		517	104			530	70		0·564		539	53			518	96		0·614	0·168
Crypt width (μm)†	115			2·9	118			3·0	0·436	113			2·9	120			2·9	0·111	0·554
Crypt volume (μm^3^)†	5·34 × 10^6^			3·51 × 10^5^	6·14 × 10^6^			3·67 × 10^5^	0·159	5·43 × 10^6^			3·60 × 10^5^	6·05 × 10^6^			3·63 × 10^5^	0·198	0·610

* Significant effect of the intervention (*P* < 0·05).

† Data are presented as LSM for data adjusted for pre-intervention measurement, age, sex, endoscopy procedure, BMI and smoking status (ANOVA general linear model (GLM)).

‡ Analysed using the non-parametric Mann–Whitney test.

### Resistant starch increased total mitoses in older participants

**Fig. 2.**
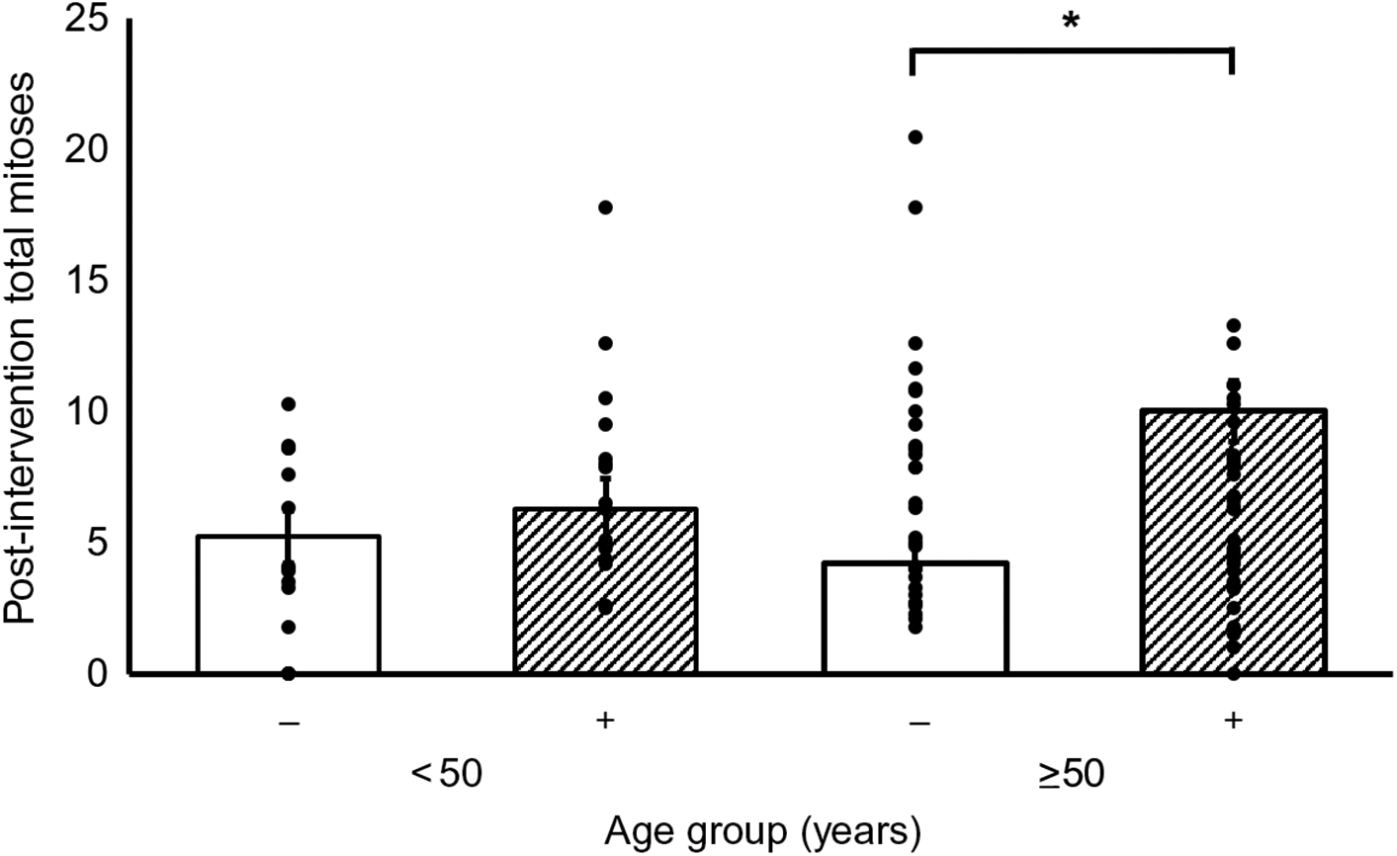
Differences in the effects of resistant starch supplementation on post-intervention total mitoses between younger (<50 years old) and older (≥50 years old) participants. Data are presented as individual data plots, and bars represent least squares means for data adjusted for pre-intervention total mitoses, age, sex, endoscopy procedure, BMI and smoking status (ANOVA general linear model (GLM)). Error bars represent standard errors of the mean. * Significant effect of the intervention (*P* < 0·05).

**Fig. 3.**
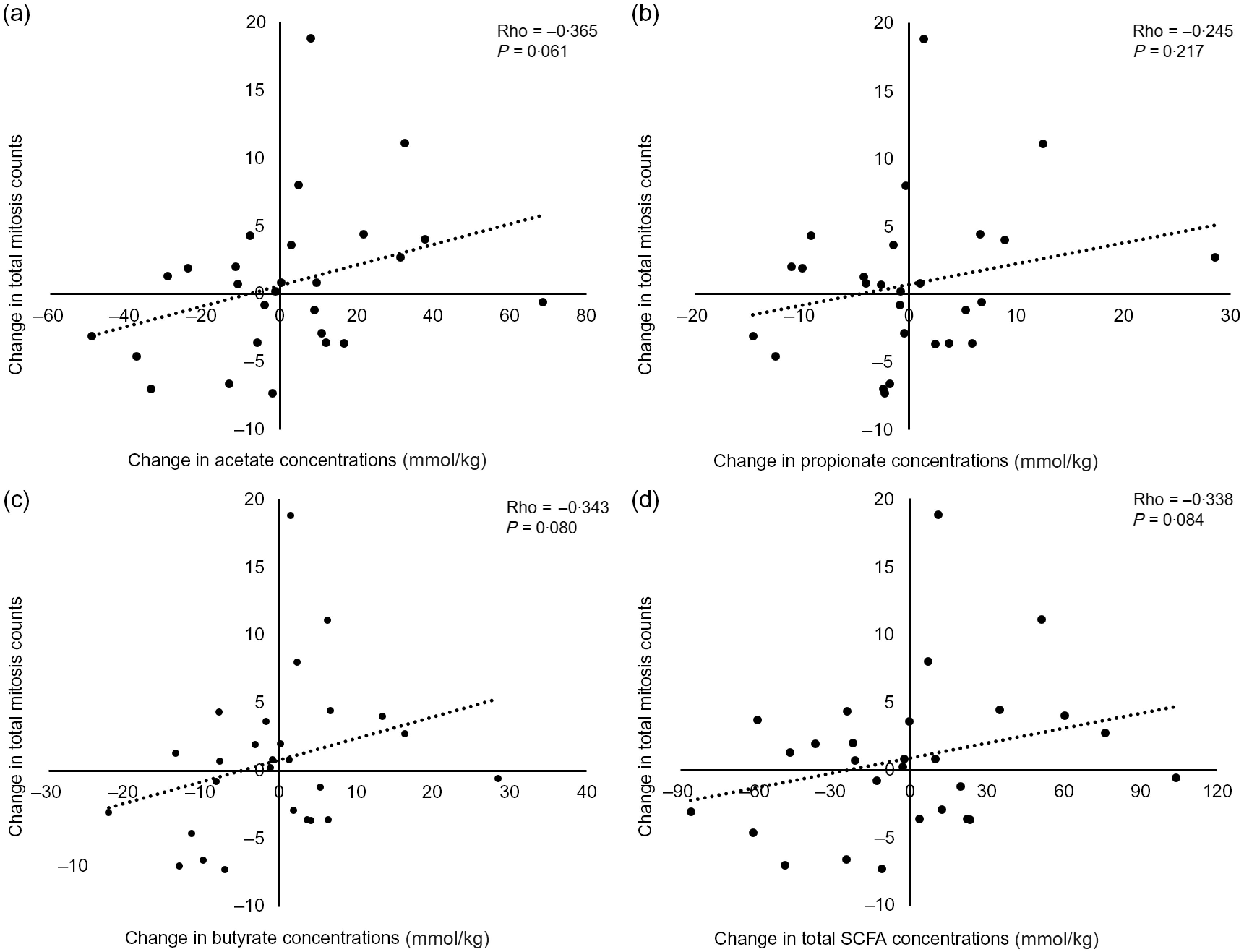
Positive relationship between changes in faecal (a) acetate, (b) propionate, (c) butyrate and (d) total SCFA concentrations and the change in total mitoses counts in Dietary Intervention, Stem cells and Colorectal Cancer Study participants supplemented with resistant starch.

When investigating the effects of NDC on total mitoses, there was a significant effect of age (*P* = 0·042) when included as a covariate. Therefore, *post hoc* analyses were performed by dividing participants into younger (aged <50 years) and older (aged ≥50 years) groups. This showed that the increase in total mitoses with RS was limited to older participants in whom supplementation with RS more than doubled the total number of mitoses compared with placebo (least squares means 10·0 *v*. 4·2) (*P* = 0·002) (Fig. 2). The distribution of mitotic cells along the crypt for those aged >50 years for both RS and placebo groups is shown in Fig. 3 in online Supplementary Material. Although RS increased the total number of proliferating cells in the crypts, there was no effect on the distribution of proliferating cells (i.e. the proportion of mitotic cells in the top half of the crypts) (*P* = 0·727). There were no statistically significant effects of PD on total mitoses in older (*P* = 0·508) or younger (*P* = 0·844) participants.

### Is the age-dependent effect of resistant starch on total mitoses due to differences in habitual dietary fibre intake or in faecal SCFA concentrations?

To test the hypothesis that the differences in the effects of RS on total mitoses between younger and older participants are associated with differences in habitual dietary fibre intake, we compared habitual dietary fibre intake between the two groups using two-sample *t* tests. There were no statistically significant differences in habitual dietary fibre intake between younger (mean 29·0 (sd 2·8) g) and older (mean 30·4 (sd 2·0) g) participants (*P* = 0·676). Furthermore, there were no statistically significant differences in habitual total starch intake between the two age groups (*P* = 0·635). The same relationship was observed for those participants given RS only (*P* = 0·320). Furthermore, there were no statistically significant relationships between habitual dietary fibre intake and CCPS markers (data not shown).

To test the hypothesis that differences in the effects of RS on total mitoses between younger and older participants are associated with differences in faecal SCFA concentrations (acetate, propionate and butyrate) at baseline, we compared pre- and post-intervention SCFA concentrations between the two age groups for all participants using independent-sample *t* tests. There were no statistically significant differences in faecal concentrations of acetate, propionate or butyrate between younger and older participants (Table 3). These analyses were repeated only in those given RS and in those given PD, and there were no within-group differences in pre- and post-intervention SCFA concentrations between younger and older participants (data not shown).

**Table 3.** Faecal SCFA concentrations in younger (<50 years) and older (≥50 years) Dietary Intervention, Stem cells and Colorectal Cancer study participants pre- and post-intervention irrespective of treatment group (Mean values with their standard errors)

	SCFA	<50 years old		≥50 years old		P*
		Mean	sem	Mean	sem	
Pre-intervention faecal SCFA concentrations (mmol/kg)	Acetate	42·9	3·4	44·5	2·7	0·705
	Propionate	13·8	1·3	13·2	0·98	0·718
	Butyrate	12·8	1·4	12·6	1·1	0·932
	Total SCFA	77·6	8·7	77·9	7·8	0·979
Post-intervention faecal SCFA concentrations (mmol/kg)	Acetate	45·6	4·7	45·0	2·8	0·919
	Propionate	15·1	2·0	12·4	0·85	0·984
	Butyrate	13·3	1·8	12·3	1·0	0·631
	Total SCFA	79·6	12·0	78·9	5·5	0·959

* Independent-samples *t* test.

We investigated relationships between change in faecal SCFA (acetate, propionate, butryate and total SCFA) concentrations as a result of the dietary intervention (post-intervention concentration minus baseline concentration) and change in the measured CCPS markers post-intervention. This revealed a trend (*P* < 0·1) for a positive correlation between change in faecal acetate concentrations and change in total mitoses (Spearman’s rho = 0·237, *P* = 0·079). This relationship was strengthened when performing the analyses only in those participants receiving the RS intervention (Spearman’s rho = 0·365, *P* = 0·061, Fig. 3), and a similar relationship was observed between change in faecal butyrate concentrations and change in total mitoses (Spearman’s rho = 0·343, *P* = 0·080, Fig. 3).

### Relationships between expression of cell cycle regulators (*CCND1, c-JUN* and *c-MYC)* and crypt cell proliferation state

We have reported previously that there was no effect of NDC supplementation on expression of the three cell cycle regulators, *CCND1*, *c-JUN* (Jun proto-oncogene) and *c-MYC*, in the rectal mucosa^(40)^. Here we have extended that analysis (using Spearman’s rank correlation) and observed no statistically significant relationships between expression of *CCND1*, *c-JUN* and *c-MYC* and measures of CCPS or crypt dimensions. This lack of relationship was evident at both pre- and post-intervention for all participants and also in those supplemented with RS only (data not shown).

### Effects of non-digestible carbohydrates and age on regulators of apoptosis

**Fig. 4.**
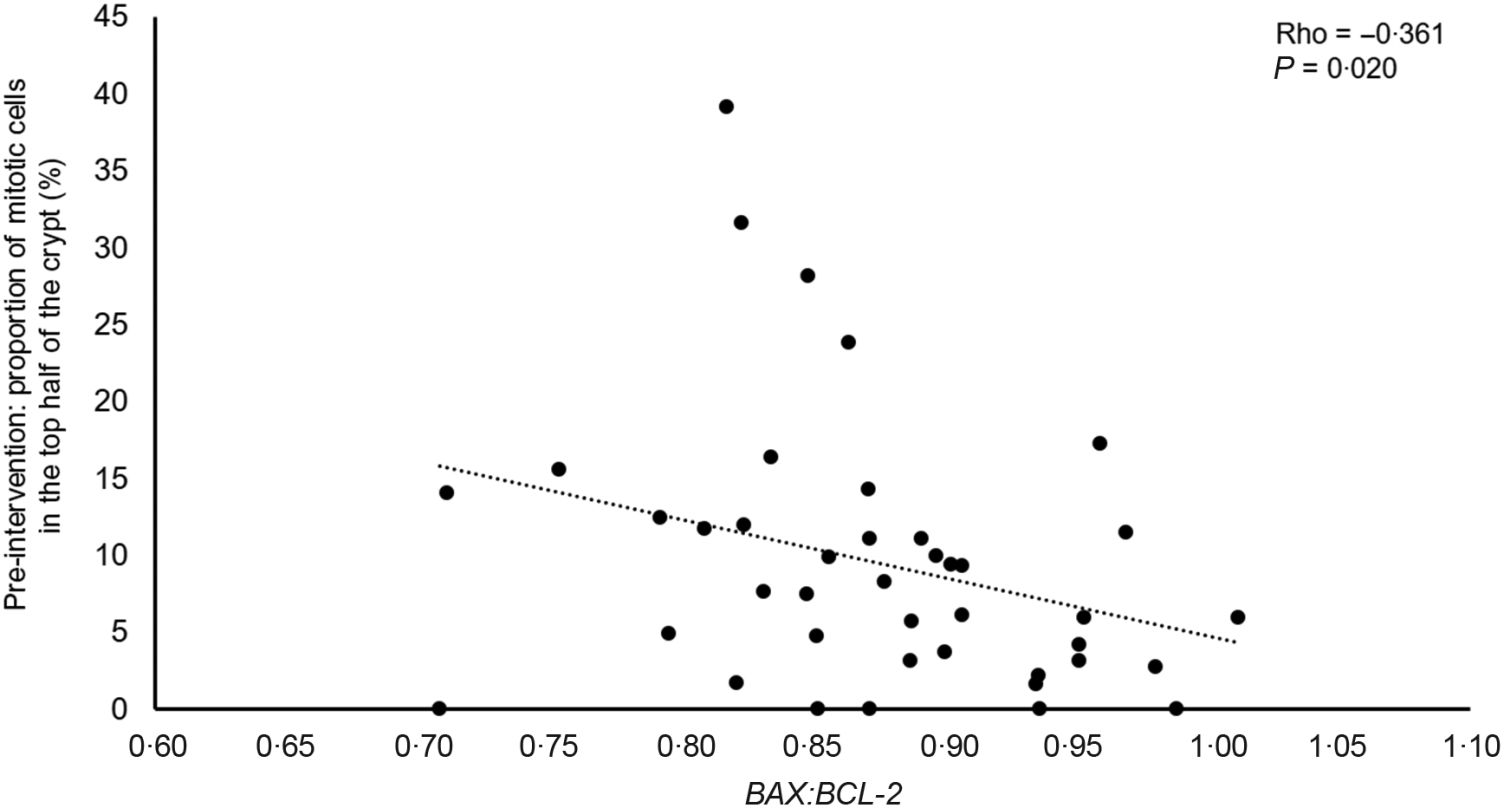
Inverse relationship between *BAX:BCL-2* and the proportion of mitotic cells in the top half of the crypt at baseline (pre-intervention). Gene expression data are presented as ratios for *BAX*:*BCL-2* expression, expressed as adjusted copies relative to the *18S* and *β2M* reference genes. *n* 41. *BAX*, BCL2 associated X; *BCL-2*, B-cell lymphoma 2.

The expression at the mRNA level of two regulators of apoptosis, *BAX* (pro-apoptotic) and *BCL-2* (anti-apoptotic), was quantified in rectal mucosal biopsies. Expression of *BAX*, *BCL-2* and the ratio *BAX:BCL-2* were not affected by supplementation with RS or PD (Table 4). We also investigated relationships between these markers of apoptosis and CCPS pre- and post-intervention. At baseline, there was a statistically significant inverse correlation between *BAX:BCL-2* and the proportion of mitotic cells in the top half of the crypt at baseline (Spearman’s rho = −0·361, *P* = 0·020), suggesting that lower levels of apoptosis were associated with a greater proportion of mitotic cells in the top half of the crypt (Fig. 4). Furthermore, *BAX* expression and age were inversely correlated (Spearman’s rho = −0·300, *P* = 0·036).

**Table 4.** Effects of resistant starch (RS) and polydextrose (PD) on expression of *BAX* and *BCL-2* mRNA* (Least squares means (LSM) with their standard errors; medians and interquartile ranges (IQR))

	Effect of RS								*P*	Effect of PD								*P*
	**–**				**+**					**–**				**+**				
	LSM	Median	IQR	sem	LSM	Median	IQR	sem		LSM	Median	IQR	sem	LSM	Median	IQR	sem	
*BAX*	2·751			0·404	2·357			0·399	0·503	2·291				2·816				0·371
*BCL-2*†		0·266	0·475			0·231	0·180		0·545		0·266	0·158			0·220	0·581		0·381
*BAX:BCL-2*	0·816			0·015	0·820			0·015	0·858	0·818			0·013	0·818			0·016	0·992

*BAX*, BCL2 associated X; *BCL-2*, B-cell lymphoma 2.

* Data are presented as LSM for data adjusted for pre-intervention measurement, age, sex, endoscopy procedure, BMI and smoking status (ANOVA general linear model (GLM)). Gene expression data at the mRNA level are expressed as adjusted copies relative to the geometric mean of *18S* and *β2M*.

† Analysed using the non-parametric Mann–Whitney test.

## Discussion

Cell proliferation and apoptosis in the large bowel mucosa are tightly regulated processes, and an imbalance between mitoses and apoptosis favouring hyper-proliferation is a characteristic of a pro-tumorigenic state^(24)^. Perhaps more important is the distribution of mitotic cells within the crypt. An abnormal distribution of mitotic cells (increased proportion of mitotic cells in the top half of the crypt) or an enlargement of the proliferative compartment is one of the earliest detectable changes in the normal mucosa prior to the development of malignancy^(25,47)^. There is convincing evidence for a protective role of NDC on the risk for developing CRC, and a plausible underlying mechanism is that this is due to effects on expression of genes that regulate cell proliferation and apoptosis. However, the evidence in both animal and human studies for the effects of specific NDC, and in particular RS, is conflicting, and effects appear to be influenced by differences in dose, type of RS and health status of study participants^(16)^.

In this study, we supplemented seventy-five healthy individuals with RS and/or PD (two NDC) or placebo for 50 d in a 2 × 2 factorial design. In these healthy individuals, RS supplementation significantly increased the total number of mitotic cells in rectal mucosal crypts from older (>50 years) participants compared with placebo. We did not observe any significant effects of PD supplementation, nor an effect of an interaction between the two NDC, on CCPS or related outcomes. To our knowledge, only one other human study has investigated the effects of PD on colorectal crypt cell proliferation. Jie *et al.* assessed cell proliferation in the caecum as an indirect measure of SCFA (mainly butyrate) production^(33)^. The authors reported higher concentrations of acetate and butyrate in stool after consumption of 4, 8 and 12 g of PD for 28 d, and they observed increased cell proliferation in the caecal mucosa, with most of this increase occurring in the bottom 60 % of the crypt^(33)^. The lack of effect of PD (or RS in our study) on numbers of proliferating cells in the upper crypt compartments suggests that these carbohydrates have no adverse effects on the colorectal mucosa and that the observed effects on proliferation in the lower compartments may be due to butyrate fuelling the colonocytes^(33)^. We tested that hypothesis in an earlier study carried out in rats^(48)^. We observed that adding haricot beans (*Phaseolus vulgaris*; a good source of fermentable fibre) to a white bread-based (low fibre) diet increased markedly the production of SCFA in the large bowel. In addition, crypt cell proliferation rate in the rat caecum increased within 24 h of the dietary change was remained elevated for at least 2 weeks. We concluded that SCFA stimulates crypt-cell proliferation in conditions of hypo-proliferation, that is, that this is a normal physiological response to the extra SCFA^(48)^. The difference in findings between this study and ours may be due to the different anatomical sites investigated (caecum *v*. rectum) or to the use of *ex vivo* *v*. *in vivo* methods for assessing cell proliferation. While the pulse labelling with [3 H]thymidine approach may have sensitivity advantages, it may be subject to artefacts as a result of the significant time in culture (4 h) after biopsy collection.

In the present study, the effect of RS appeared to be limited to individuals aged ≥50 years, and was not attributable to differences in (i) habitual fibre intake, (ii) faecal butyrate (or other SCFA; acetate and propionate) concentrations or (iii) baseline levels of cell proliferation between younger and older participants. Faecal SCFA may not be fully representative of intraluminal SCFA concentrations. For example, a study in human sudden death victims reported a reduction in SCFA concentrations from the proximal to the distal colon and reported similar total SCFA concentrations in the sigmoid and rectum to those reported in faecal samples^(49)^. Furthermore, a limitation of this study is that we do not have data on stool output. Since stool bulk may affect the concentration of its contents such as SCFA, an increase in stool bulk (expected after consumption of RS and PD) could mask increases in SCFA concentrations.

Few human intervention studies have investigated the effects of supplementation with RS on CCPS and some of these studies have been performed in individuals at greater risk of CRC, for example, those with adenomas or familial adenomatous polyposis (FAP)^(50)^, or in CRC patients^(32)^ as distinct from healthy individuals (Table 5). In a randomised controlled trial with 206 FAP patients, Burn *et al.*^(31)^ reported 28 % higher mitoses in those randomised to RS, but this effect was not significant (*P* = 0·12). In sixty-five CRC patients, supplementation with 30 g of RS/d, in the form of a 1:1 blend of Novelose 240 (RS type 2) and Novelose 330 (RS type 3), for up to 4 weeks resulted in a lower proportion of mitotic cells in the top half of the crypt^(32)^. An early study reported a small reduction in total mitoses in fourteen healthy participants supplemented with high-amylose maize starch (62 % RS) for 2 weeks^(17)^. In contrast, two studies found no effect on cell proliferation after supplementation with RS type 2 in healthy participants^(28,29)^. In male c57bl/J mice, long-term supplementation (up to 18 months) with high-amylose maize starch resulted in increased cell proliferation^(52)^. Although there were no significant effects on total levels of mitoses, potato starch (RS type 2) supplementation for 19 d increased cell proliferation in middle and luminal compartments of colonic crypts in male pigs^(53)^.

**Table 5.** Summary of findings from human studies investigating the effects of resistant starch (RS) on cell proliferation in the large bowel (Medians and ranges)

Study	*n* (males/females)	Health status	Mean age (years)		Source of RS	RS dose (per d)	Intervention duration	Effect of RS on cell proliferation
			Median	Range				
van Munster *et al.* (1994)^(51)^	14 (9/5)	Healthy		28–73	High-amylose maize starch (Hylon VII, RS type 2)	45 g	2 weeks	Decreased
Humphreys *et al.* (2014)^(39)^	23 (17/6)	Healthy		50–75	Butyrylated high-amylose maize starch (+ high-red meat diet)	40 g (+ 300 g red meat)	4 weeks	Restored (decreased) cell proliferation levels to baseline, when given second following high-red meat diet
Wacker *et al.* (2002)^(28)^	12 (7/5)	Healthy	25		High-amylose maize starch (Hylon VII, RS type 2)	Males 59·7 g and females 50·7 g	4 weeks	No effect
Worthley *et al.* (2009)^(29)^	17 (11/6)	Healthy	60		High-amylose maize starch (Hi-maize 958, RS type 2)	25 g	4 weeks	No effect
Present study	75 (40/35); 35 given RS	Healthy	52		High-amylose maize starch (Hi-maize 260, RS type 2)	23 g	7 weeks	Increased (in older participants)
Dronamraju *et al.* (2009)^(32)^	62 (35/27)	Colorectal cancer	66		1:1 Blend of Novelose 240 (RS type 2) and Novelose 330 (RS type 3)	30 g	<4 weeks	Reduction in the proportion of mitotic cells in the top half of the crypt
Grubben *et al.* (2001)^(22)^	23 (12/11); 13 given RS	Recently removed adenomas	57		High-amylose maize starch (Hylon VII, RS type 2)	45 g	4 weeks	No effect
van Gorkom *et al.* (2002)^(21)^	86 (53/30); 28 given RS	≥1 Sporadic adenoma(s) removed within 5 years	60		High-amylose maize starch (Hylon VII, RS type 2)	30 g	8 weeks	No effect
Burn *et al.* (2011)^(50)^	131 (65/66); 61 given RS	Familial adenomatous polyposis	18		1:1 Blend of potato starch and high-amylose maize starch (Hi-maize 958, RS type 2)	30 g	1–12 years	No effect

The differences in the findings in CRC patients, or those at greater risk of CRC, may result from differences in baseline levels of cell proliferation. For example, mean baseline crypt mitotic cell count in our healthy participants was 6·8, whereas in the CRC patients reported by Dronamraju *et al.*^(32)^ baseline levels of proliferation were about 40 % higher. In healthy participants, high red meat consumption for 4 weeks increased cell proliferation in the rectal mucosa by two-thirds, and this was restored to levels similar to baseline (approximately seven positive cells per crypt) with butyrylated RS supplementation^(39)^.

Since differences in crypt cell kinetics may be due to changes in expression of genes regulating cell proliferation and/ or apoptosis, we investigated relationships between expression of three cell cycle regulators, *CCND1*, *c-JUN* and *c-MYC*, in the rectal mucosa and then measured CCPS markers (and crypt dimensions), but found no statistically significant correlations. For the same DISC Study participants, we reported previously no effects of supplementation with NDC on expression of these three genes^(40)^. In the current investigation, we found no effect of RS or PD on expression of two regulators of apoptosis, *BAX* and *BCL-2*, in the healthy colorectal mucosa. Very few previous studies have investigated the effects of NDC on apoptosis by quantifying the expression of *BAX* or *BCL-2* in the colorectal mucosa. In mouse models of CRC, supplementation with RS type 3^(54)^ and with Indica rice RS^(38)^ reduced Bcl-2 and increased Bax expression. At baseline in the present study, *BAX:BCL-2* ratio correlated inversely with the proportion of mitotic cells in the top half of the crypt, suggesting lower proportions of mitotic cells in the top half of the crypt with greater levels of apoptosis. Furthemore, *BAX* expression and age were negatively correlated (data not shown), in agreement with observations in adenocarcinoma patients aged >50 years^(55)^. Since lower apoptosis, which may result from reduced *BAX* expression or reduced *BAX:BCL-2* ratio, favours hyper-proliferation and tumorigenesis, this could be a potential mechanism for the effect of age on CRC risk. Interestingly, our findings suggest that the effects of RS on CCPS may be age-dependent, which is of particular importance as age is the strongest risk factor for CRC.

In conclusion, RS supplementation increased cell proliferation in the crypts of the macroscopically-normal rectum of healthy individuals, and these effects were restricted to older participants (≥50 years). Although there have been few similar studies, this effect was unexpected since most previous studies in healthy people have shown no effect^(28,29)^ or reduced cell proliferation in the large bowel^(51)^ with RS consumption. However, in the normal colorectal epithelium, butyrate, produced from RS, is the primary energy source for colonocytes and contributes to the regulation of cell proliferation rates, which may explain the observed promotion of mitosis^(48,56)^. Our findings suggest that different sources of dietary fibre (we compared RS and PD) have different effects on crypt cell dynamics in human large bowel mucosa. However, since we found no change in the proportion of mitotic cells in the top half of the crypt in the healthy individuals in the present study, it seems unlikely that these effects of RS have any implications for CRC risk. This study provides no support for the idea that fibre-supplemented foods may increase CRC risk^(57)^.

## Supplementary material

For supplementary material accompanying this paper visit https://doi.org/10.1017/S0007114520001312. click here to view supplementary material
