# Supplementary material for: Resistant starch supplementation increases crypt cell proliferative state in the rectal mucosa of older healthy participants
Source: Br J Nutr. 2020 Apr 13;124(4):374–85. doi: 10.1017/S0007114520001312 (PMC7369377; doi:10.1017/S0007114520001312)
Supplement: Supplementary file 1 [file S0007114520001312sup001.docx]

**Supplementary Material**


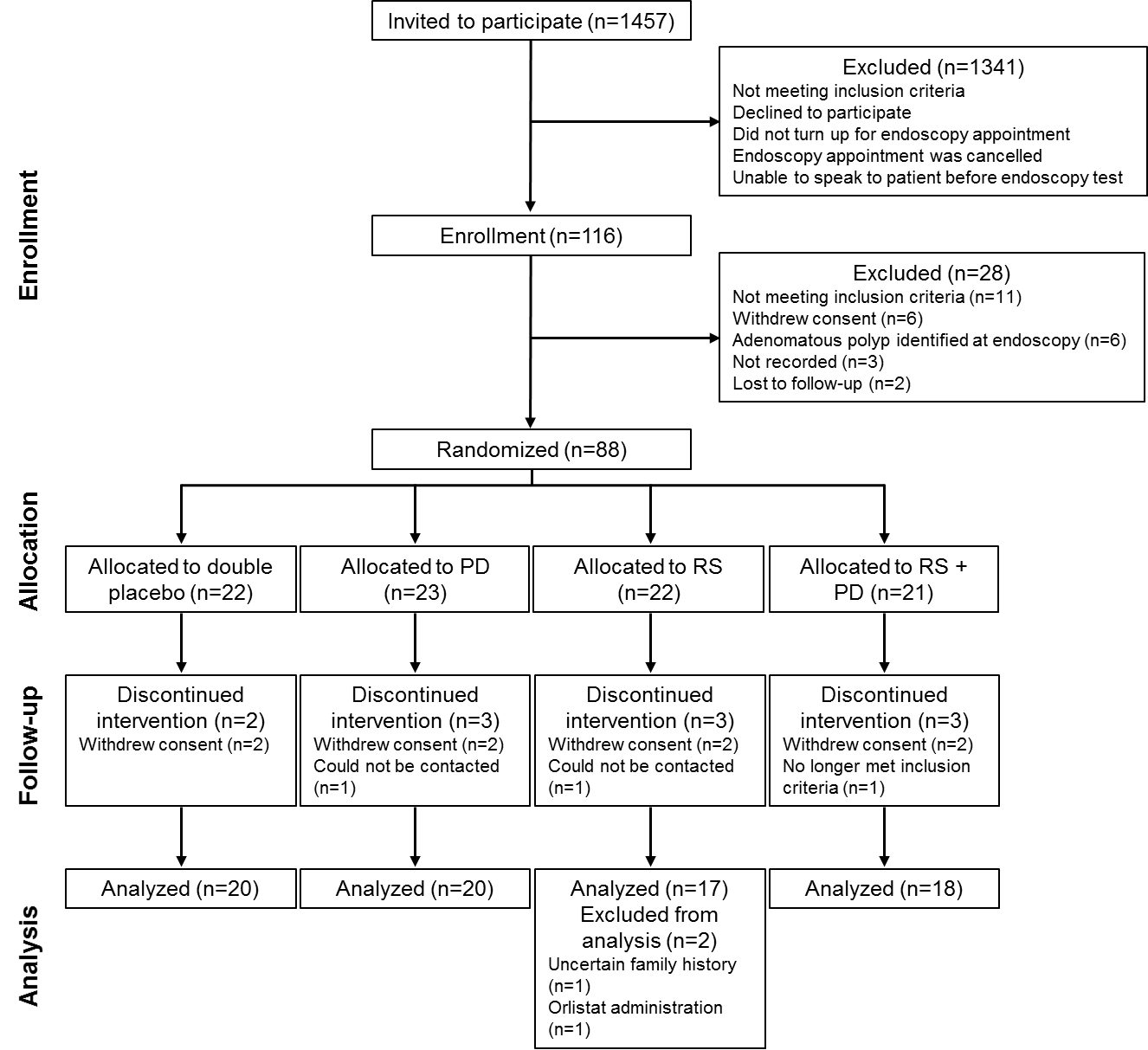


**Supplementary Figure 1: Consolidated Standards of Reporting Trials Diagram: flow of participants through the Dietary Intervention, Stem cells and Colorectal cancer Study randomized controlled trial.** PD, polydextrose; RS, resistant starch.

**
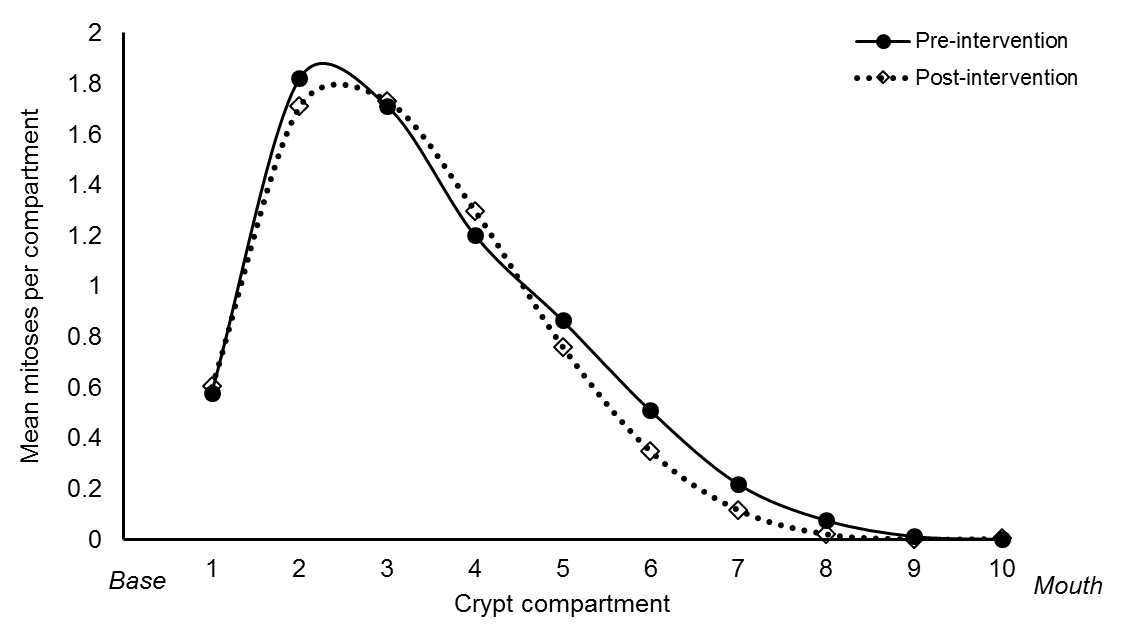
**

**Supplementary Figure 2: Mean number of mitoses per crypt compartment pre- and post-intervention for all DISC Study participants**

**
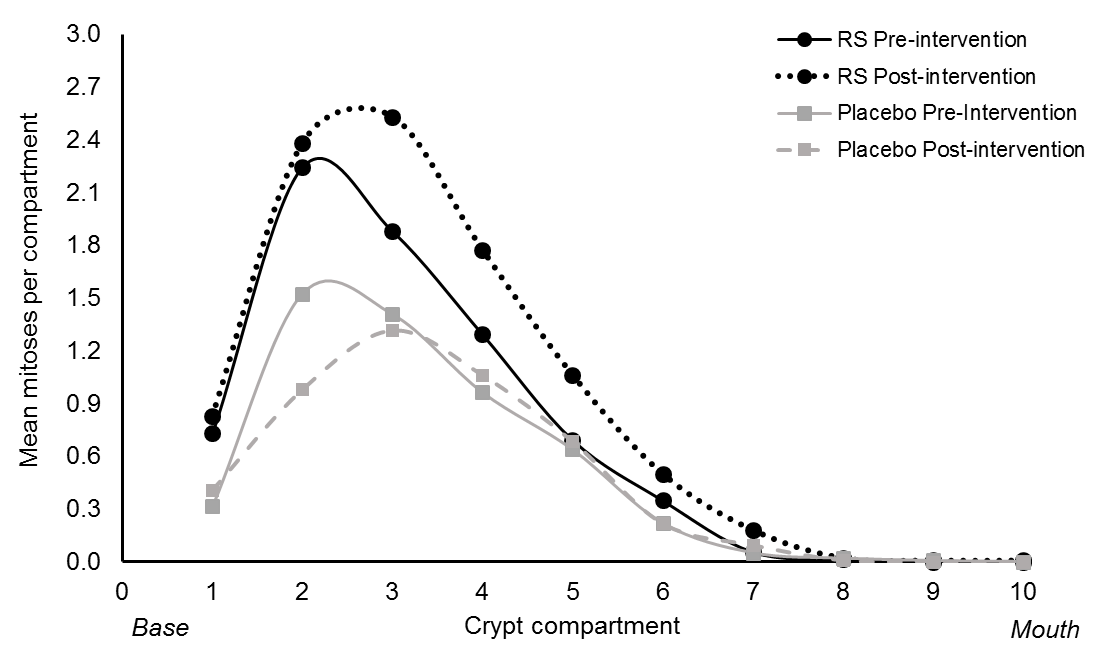
**

**Supplementary Figure 3: Mean number of mitoses per crypt compartment pre- and post-intervention for all older (>50 years old) DISC Study participants given RS or placebo**

Supplementary Methods:

Supplementary Table 1: Primer sequences for quantification of *CCND1, c-MYC* and *SFRP1* by qPCR

| **Gene** | **Forward primer sequence** | **Reverse primer sequence** |
| --- | --- | --- |
| *18S* | GGCTCATTAAATCAGTTATGGTTCCT | GTATTAGCTCTAGAATTACCACAGTTATCC |
| *B2M* | AAAGATGAGTATGCCTGCCGT | ACTTAACTATCTTGGGCTGTGACAA |
| *CCND1* | TTGTACCTGTAGGACTCTCATTCG | ACAGCACTGTGAGCTGGCT |
| *c-MYC* | AGATCCGGAGCGAATAGGG | GTCCTTGCTCGGGTGTTGTA |
| *SFRP1* | TGGTGTGGATCTATTGGCTG | TCACTTTCTGGGCTTGACCT |
